# Association Between Sensory Impairment and Dementia: The Roles of Social Network and Leisure Activity

**DOI:** 10.3233/JAD-230041

**Published:** 2023-07-18

**Authors:** Christina S. Dintica, Amaia Calderón-Larrañaga, Davide Liborio Vetrano, Weili Xu

**Affiliations:** a Aging Research Center, Department of Neurobiology, Care Sciences and Society, Karolinska Institutet and Stockholm University, Stockholm, Sweden; b Department of Psychiatry and Behavioral Sciences, University of California, San Francisco, San Francisco, CA, USA; c Stockholm Gerontology Research Center, Stockholm, Sweden

**Keywords:** Alzheimer’s disease, dementia, hearing impairment, sensory impairment, vision impairment

## Abstract

**Background:**

Evidence suggests that sensory impairment is linked to dementia; however, the role of social network and leisure activity in this relationship is unclear.

**Objective:**

Examine the association of hearing and visual impairment with dementia, and whether a rich social network and leisure activity moderates this association.

**Methods:**

Dementia-free older adults from the Swedish National Study on Aging and Care in Kungsholmen (*n* = 2,579) were followed up for up for a median of 10 years (interquartile range = 6). Visual impairment was assessed with a reading acuity test, and hearing impairment was ascertained via self-report and medical records. Dementia was diagnosed following international criteria. Data on social network and leisure activity was collected via self-report. Hazard ratios (HRs) of dementia risk were derived from Cox regression models.

**Results:**

Dual impairment, but not single impairment in hearing and vision was associated with a higher risk of dementia (HR: 1.62, 95% CI: 1.16 to 2.27). Compared to participants with no sensory impairment and a moderate-to-rich social network, those with dual impairment and low social network or leisure activity had higher dementia risk (HR: 2.08, 95% CI: 1.43 to 3.22; HR: 2.08, 95% CI: 1.43 to 3.22, respectively), whereas participants with dual impairment with a moderate-to-rich social network or leisure activity did not have significantly higher dementia risk (HR; 1.42, 95% CI: 0.87 to 2.33; HR; 1.42, 95% CI: 0.87 to 2.33, respectively).

**Conclusion:**

A richer social network and participation in stimulating activities may mitigate the higher dementia risk in older adults with dual impairment in vision and hearing.

## INTRODUCTION

Impairments in hearing and vision are frequent in older adults, with an estimated 50% of individuals over 60 years reporting hearing or vision loss and 11.3% of those over the age of 80 reporting having both, referred to as dual impairment [1, 2]. These impairments will affect a growing proportion of the population due to increased longevity [3]. Moreover, sensory impairments have been linked to increased mortality and functional problems [4–6].

Several prospective studies have shown that hearing and visual impairments in older adults increase the risk of cognitive impairment and dementia independently, with some inconsistent results [7–11]. The majority of previous studies have focused on single impairments in hearing or vision, thus the association between combined hearing and visual impairment, or dual impairment, on dementia risk remains unclear. Studies investigating the coexistence of both impairments have reported greater risk of health outcomes such as functional decline and mortality than a single sensory impairment [12, 13]. Importantly, dual sensory impairments may also increase the risk of cognitive impairment, more so than the presence of only one impairment [13–15]. However, findings have been inconsistent, with others reporting no added risk of cognitive impairment related to dual sensory impairments [16].

Growing evidence suggests that engagement in mentally, physically, or socially stimulating leisure activities is associated with lower dementia risk in older adults [17–20]. Furthermore, various indicators of a strong social network, such as social connections or social support, have been linked to a reduced risk of dementia both independently and in conjunction with a higher level of participation in leisure activities [17, 18, 21]. Additionally, epidemiological research supports the idea that the relationship between sensory impairments and dementia is influenced by the strong correlation between sensory impairments and social isolation, loneliness, and reduced involvement among older adults [22–24] with some reports suggesting social isolation in particular may be a mediating factor between hearing loss and cognitive decline [25]. However, another study found that use of hearing aids improved cognitive function independently of depression and social isolation [26], hence the role of leisure activity and social engagement in the sensory impairment-dementia association remains unknown.

In the present study, we aimed to examine the association of hearing and vision impairment with dementia, and to explore whether a rich social network and engagement in leisure activities may attenuate dementia risk related to sensory impairment.

## METHODS

### Study population

**Fig. 1:**
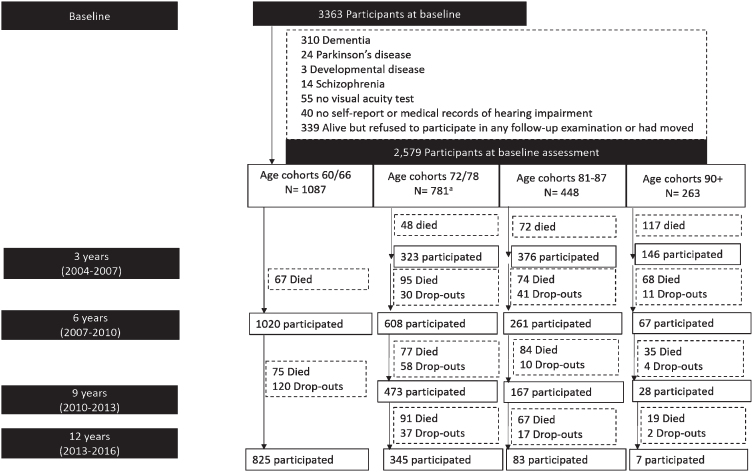
Flowchart of study participants. The age-cohort 78 included 371 participants who were re-assessed at 3 years follow-up, whereas for the *n* = 410 participants in the age cohort 72 years the first follow-up was after 6 years, based on the SNAC-K study design.

The Swedish National Study on Aging and Care-Kungsholmen (SNAC-K) is an ongoing prospective population-based study [27], including 3363 people aged≥60 years recruited at baseline (March 2001–June 2004), either community dwelling or in institutions in Kungsholmen (central Stockholm, Sweden). Recruitment was done through age-stratification, with “young” age-cohorts (60, 66, and 72) followed-up every sixth year and “old” age-cohorts (⩾78 years) every third year, due to faster health changes and more attrition in older age. We excluded people with prevalent dementia (*n* = 310), Parkinson’s disease (*n* = 24), schizophrenia (*n* = 13) or developmental disorders (*n* = 3), and missing information on hearing (*n* = 40), or vision (*n* = 55) assessments at baseline. We further excluded 339 individuals who failed to participate in at least one follow-up. Thus, 2579 participants were included in the current study (Fig. 1). During the 12-year follow-up, 1,062 people (41%) died and 320 (12.4%) were lost to follow-up (participation rate = 87.6%) (Fig. 1)

The ethical committee at Karolinska Institutet and the regional ethical review board in Stockholm approved all parts of SNAC-K, including linkage with registries. All participants or their next-of-kin (in case of cognitively impaired participants) provided written informed consent.

### Assessment of sensory impairment

#### Vision impairment

The Jaeger eye chart was used to assess near visual acuity at a normal reading distance. If the participant typically wears eyeglasses or contact lenses full-time, they were instructed to wear them during the test. The “J” value of the smallest block of text the participant could read was recorded (J1 being the smallest and J7 the largest). Each eye was tested separately and the average of the two values was used. A J1 value corresponds to a 20/20 (normal vision) [28]. Hence, impaired vision was defined as reading acuity corresponding to a J2 value or higher.

#### Hearing impairment

This was assessed during the nurse interview at baseline based on self-reported information on hearing ability. The participants were asked “Do you have trouble hearing?” with possible answers *a) Yes, but uses hearing aid; b) Yes, but uses no aid; and c) No*. Moreover, medical records from the national patient registry (NPR) which covers all inpatient and outpatient specialist care in Sweden, were obtained through linkage and the following ICD codes were included in hearing loss/deafness: H80 Otosclerosis; H90 Conductive and sensorineural hearing loss; H911 Presbycusis; H913 Deaf mutism, not elsewhere classified; H919 Hearing loss, unspecified; Q16 Congenital malformations of ear causing impairment of hearing; Z453 Adjustment and management of implanted hearing device; Z461 Fitting and adjustment of hearing aid; Z962 Presence of otological and audiological implants; and Z974 Presence of external hearing-aid. We categorized participants as hearing impaired if they either reported self-reported hearing impairment or had a hearing loss/deafness ICD codes as described above.

Dual impairment was defined as having both vision impairment and hearing impairment.

### Leisure activities

During the baseline nurse interview, participants were asked about their engagement in 26 leisure activities over the past 12 months (Supplementary Material 1). These activities were categorized as mental, social, or physical and the level of engagement was recorded as low, moderate, or high [17]. A leisure activity index was created by summing the three types, with scores ranging from 0 to 6. The level of engagement in mental activity was coded as low (1 or more), moderate (2 to 3 activities), or high (4+ activities). Level of engagement in social activity was coded as low (0 activities), moderate (1 activity), or high (2+). Level of engagement in physical activity was coded as low (performed less than once/week), moderate (performed at least once/ week), or high (performed more than once/week). The three types of activities were summed to create a leisure activity index (range 0–6), and level of engagement was coded as low (scores 0–1), moderate (scores 2–3), or high (scores 4–6).

### Social network

An index of late-life social network was created on the basis of network size (marital status, living arrangements, number of children, and frequency of contacts) and perceived social support (satisfaction with contacts, perceived support, and sense of affinity and belonging to various groups) (Supplementary Material 2) [17]. These two measures were standardized and summed and to create social network index with three levels (tertiles).

### Dementia diagnosis

Dementia was diagnosed according to the Diagnostic and Statistical Manual of Mental Disorders-IV-TR (DSM-IV-TR) criteria, using a validated three-step procedure. Two independent physicians carried out preliminary clinical diagnoses by reviewing the participant’s neurological, cognitive, and physical status. In case of disagreement an external neurologist made the final diagnosis [17]. Physicians also identified dementia cases among deceased participants by consulting death certificates and medical records, when available.

### Covariates

Education was categorized as elementary, professional schools, high school, or university. Smoking status was dichotomized as never versus current/former and alcohol consumption as no/occasional versus light-to-heavy drinking. Medical conditions (including heart disease, cerebrovascular disease, and diabetes) were diagnosed using the International Classification of Diseases, Tenth Revision (ICD-10) on the basis of clinical examination, self-report, medication use, or linkage with the NPR [29]. Depression was diagnosed according to the DSM-IV-TR [17]. Disability was assessed by means of the number of limitations in Activities of Daily Living (ADL) at baseline. DNA was obtained from peripheral blood samples and Apolipoprotein E (*APOE*) genotyping was performed using MALDI-TOF analysis on the Sequenom MassARRAY platform [30]. Because very few individuals carried two 4 alleles, the APOE (rs429358) polymorphism was analyzed as a binary variable, i.e., ‘4 versus no 4’.

### Statistical analysis

To compare the baseline characteristics among different groups of participants, the *χ*^2^ test was used.

Incidence rates (IRs) of dementia per 1000 person-years and 95% confidence intervals (CIs) were estimated across sensory status (no impairment, only visual impairment, only hearing impairment, and dual impairment). Follow-up time was estimated as the time from study entry until dementia, death (determined based on the Swedish Cause of Death Registry and medical records at hospital discharge), or the last SNAC-K examination. Cox models were adjusted for age, sex, and education in Model 1, and additionally by smoking, alcohol consumption, diabetes, cardiovascular disease, cerebrovascular disease, ADL limitations, and depression in Model 2. No violation of the proportional hazard assumption was observed according to the Schoenfeld residuals test.

We investigated whether social network or leisure activity levels mitigated the sensory impairment-associated dementia risk. First, interactions between sensory impairment and social network and leisure activities were tested separately, by estimating the relative excess risk due to interaction (RERI, additive interaction) and incorporating the cross-product of sensory impairment with social network and leisure activity (multiplicative interaction) in Cox regression models [31]. Second, to assess the moderating effect of social network and high leisure activity on sensory impairment-related dementia risk, we created an indicator variable, combining sensory impairment status (none, only hearing impairment, only vision impairment, dual impairment) with levels of leisure activities (low versus moderate to high) and social network (poor versus moderate to rich). Lastly, we calculated the population attributable fraction for the association between levels of social network and leisure activity and dementia in participants with sensory impairment.

A sensitivity analysis was carried out to address possible reverse causation related to prodromal dementia. Hence, participants with MMSE < 27 at baseline and/or those diagnosed with dementia in the first 3 years of follow-up were excluded (Supplementary Material 3).

All analyses were performed using Stata SE, version 15.0 (StataCorp LP., College Station, TX, USA).

## RESULTS

### Characteristics of study population

At baseline, there were 1,304 (50.6%) participants with no impairment; 426 (16.5%) with only visual impairment; 539 (20.9%) with only hearing impairment; and 310 (12.0%) with dual impairment. Participants with any sensory impairments were more likely to be older, of female sex, have lower education, have cardiovascular disease, and cerebrovascular disease, have at least one ADL limitation, consume less alcohol, and have a poor social network and low activity level (Table 1).

**Table 1:** Baseline characteristics of the study sample by sensory impairment status (*N* = 2,579)

Characteristic	No impairment *n* = 1,304 (50.6%)	Only visual impairment *n* = 426 (16.5%)	Only hearing impairment *n* = 539 (20.9%)	Dual impairment *n* = 310 (12.0%)	*p*
Age					<0.001
60 to 71	691 (53.0)	130 (30.5)	220 (40.8)	46 (14.8)
72 to 80	390 (29.9)	129 (30.3)	186 (34.5)	72 (24.5)
81 to 89	171 (13.1)	104 (24.4)	99 (18.4)	74 (23.9)
90+	52 (3.99)	63 (14.8)	34 (6.3)	114 (36.8)
Female sex	821 (63.0)	278 (65.3)	305 (56.6)	208 (67.1)	0.006
Education					<0.001
Primary	147 (11.3)	82 (19.3)	73 (13.5)	80 (25.8)
Secondary	648 (49.7)	229 (53.8)	252 (46.8)	167 (53.9)
University	509 (39.0)	115 (27.0)	214 (39.7)	62 (20.0)
Current/former smokers	717 (55.1)	213 (50.5)	295 (55.1)	150 (48.5)	0.088
Alcohol consumption
Never/occasional	351 (26.9)	184 (43.4)	147 (27.4)	149 (48.5)	<0.001
Light/moderate	708 (54.3)	169 (39.9)	303 (56.4)	121 (39.4)
Heavy	244 (18.7)	71 (16.8)	87 (16.2)	37 (12.1)
Diabetes	104 (8.0)	41 (9.6)	50 (9.3)	34 (11.0)	0.336
Cardiovascular disease*	235 (18.0)	134 (31.5)	121 (22.5)	131 (42.3)	<0.001
Cerebrovascular disease	59 (4.5)	47 (11.0)	33 (6.1)	27 (8.7)	<0.001
Any *APOE* *ɛ*4	352 (28.1)	114 (28.7)	164 (31.9)	65 (24.3)	0.147
Depression	91 (7.0)	39 (9.2)	57 (10.6)	24 (7.7)	0.065
≥1 ADL limitation	22 (1.7)	23 (5.4)	17 (3.2)	37 (11.9)	<0.001
Social network
Low	299 (23.8)	167 (42.7)	143 (27.5)	137(48.9)	<0.001
Moderate	435 (34.7)	119 (30.4)	188 (36.2)	92 (32.9)
Rich	52 (41.5)	105 (26.9)	189 (36.4)	51 (18.2)
Leisure activity					<0.001
Low	293 (22.5)	127 (29.8)	155 (28.8)	88 (28.4)
Moderate	579 (44.4)	139 (32.6)	210 (39.0)	87 (29.1)
High	331 (25.4)	64 (15.0)	122 (22.6)	34 (11.0)

^*^Includes heart failure, coronary heart disease, arrhythmia, and atrial fibrillation. Data are presented as numbers (%). Proportions were compared with *Ξ*^2^ test. *APOE* *ɛ*4, apolipoprotein *ɛ*4 allele; MMSE, Mini-Mental State Examination Missing data: *APOE* *ɛ*4 = 149, smoking = 12, education = 1, alcohol consumption = 8, ADL limitations = 2, social network index = 134, leisure activity index = 350.

**Table 2:** Incidence rates (IR) per 1000 person-years and hazard ratios (HR) with 95% CI of incident dementia (*n* = 381) by sensory impairment status (*N* = 2,579)

Visual and hearing impairment	No. participants	No. dementia cases/person-year	IR (95% CI)	HR (95% CI)^a^	HR (95% CI)^b^
None	1,304	154/12,070	12.76 (10.90 to 14.94)	Reference	Reference
Any	1,275	224/9,819	22.81 (20.01 to 26.0)	1.36 (1.09 to 1.72)	1.22 (0.97 to 1.55)
Vision only	426	69/3,184	21.67 (17.11 to 27.43)	1.27 (0.91 to 1.76)	1.12 (0.81 to 1.57)
Hearing only	539	76/4,795	15.85 (12.66 to 19.85)	1.15 (0.86 to 1.54)	1.11 (0.83 to 1.48)
Dual	310	79/1,839	42.95 (34.45 to 53.54)	1.93 (1.40 to 2.66)	1.62 (1.16 to 2.27)

^a^Model 1, adjusted for baseline age, sex, and education. ^b^Model 2, adjusted additionally for smoking, alcohol consumption, ADL limitations, diabetes, depression, cardiovascular disease, and cerebrovascular disease.

### Sensory impairment and dementia

During follow-up (median = 10.9 [interquartile range, 5.6–11.6] years), 378 people (14.6%) developed dementia (IR = 17.3 cases per 1000 person-years; 95% CI 15.6–19.1). In multi-adjusted Cox regression models, while vision and hearing impairment alone did not confer a higher dementia risk, dual impairment was associated with an 62% increased risk (HR: 1.62, 95% CI: 1.16 to 2.27), 95% CI: 0.97 to 1.55. Any impairment (dual or single) was not associated with a higher dementia risk (HR: 1.22, 95% CI: 0.97 to 1.55).

We further conducted stratified analyses by *APOE* *ɛ*4 status; any impairment, vision impairment, and dual impairment were significantly associated with a higher risk of dementia, only in *APOE* *ɛ*4 non-carriers (HR: 1.69, 95% CI: 1.24 to 2.29 in *ɛ*4 non-carriers versus HR: 0.81, 95% CI: 0.55 to 1.19 in *ɛ*4 carriers) (Supplementary Table 2). We also stratified by baseline age over or below 78; any impairment was associated with a higher dementia risk only in older participants (≥78); however, dual impairment was associated with a higher hazard regardless of age group, but more prominent in the younger participants (Supplementary Table 3).

### Moderating effect of social network and leisure activity on dementia risk

Moderate to high levels of social network and leisure activity were associated with a decreased dementia risk (Supplementary Table 1). The trend was statistically significant for both factors (*p_*trend*_* < 0.001), therefore in subsequent analyses, we merged the moderate and high tertiles of social network and leisure activity into a single category of “moderate-to-rich.” Moreover, as neither hearing or vision impairment alone were associated with a higher dementia risk, we combined these into the category “single sensory impairment” in subsequent analyses.

We further assessed whether social network or leisure activity mitigated the sensory impairment-associated dementia risk. We found no statistically significant multiplicative (HR for cross-product=1.08 [95% CI 0.86–1.35]) or additive (RERI=–0.15, *p* = 0.574) interactions between sensory impairment and social network in relation to dementia, or for sensory impairment and leisure activity (HR for cross-product=1.02 [95% CI 0.81–1.29]; RERI=–0.29, *p* = 0.333).

**Fig. 2:**
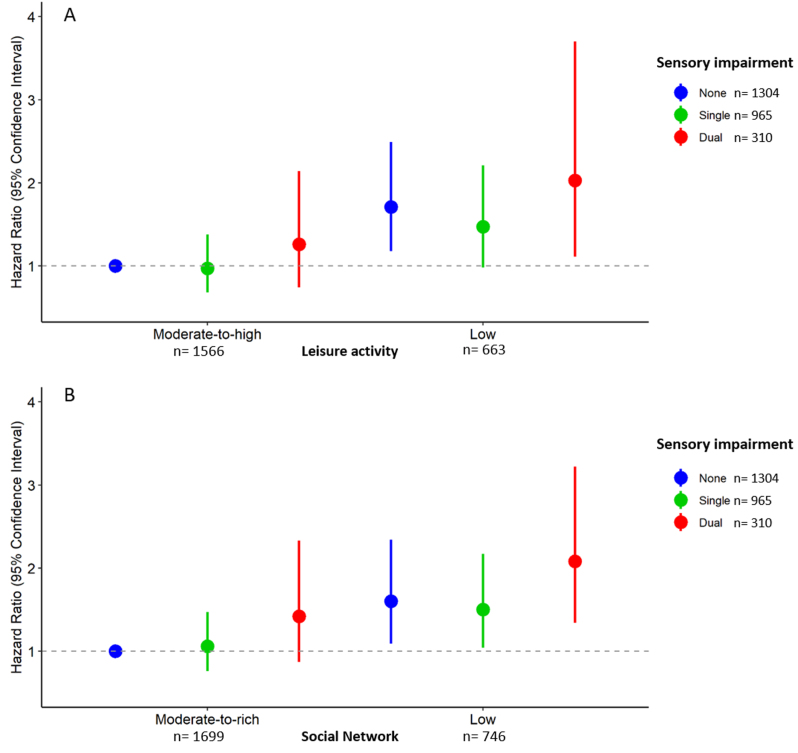
Hazard ratios (HR) with 95% CI of incident dementia (*n* = 378) by combinations of sensory impairment status and leisure activity or social network (*N* = 2,579). A) Hazard ratios (HR) with 95% CI of incident dementia (*n* = 378) by combinations of sensory impairment status and leisure activity. B. Hazard ratios (HR) with 95% CI of incident dementia (*n* = 378) by combinations of sensory impairment status and social network. Single sensory impairment includes either visual or hearing impairment, dual impairment refers to both visual and hearing impairment. Models adjusted for baseline age, sex, education, smoking, alcohol consumption, ADL limitations, diabetes, depression, cardiovascular disease, and cerebrovascular disease.

Moderate-to-high levels of social network and leisure activity both reduced the risk of dementia in participants with single or dual impairment (Fig. 2). Compared to participants with no sensory impairment and a moderate-to-rich social network, those with a single impairment and low social network as well as those with dual impairment and low social network had higher dementia risk (HR: 1.50, 95 CI: 1.04 to 2.17; HR: 2.08, 95% CI: 1.43 to 3.22, respectively), whereas participants with single or dual impairment with a moderate-to-rich social network did not have significantly higher dementia risk (HR: 1.06, 95% CI: 0.77 to 1.47; HR; 1.42, 95% CI: 0.87 to 2.33). Compared to participants with no sensory impairment and a moderate-to-high leisure activity engagement, those with a single impairment and low leisure activity as well as those with dual impairment and low leisure activity had higher dementia risk (HR: 1.50, 95 CI: 1.04 to 2.17; HR: 2.08, 95% CI: 1.43 to 3.22, respectively), whereas participants with single or dual impairment with a moderate-to-high leisure activity did not have significantly higher dementia risk (HR: 1.06, 95% CI: 0.77 to 1.47; HR; 1.42, 95% CI: 0.87 to 2.33). In participants with any sensory impairment, the population attributable fraction of moderate-to-rich social network was 0.22 (95% CI 0.03–0.38). Similarly, the population attributable fraction of moderate-to-high leisure activity was 0.25 (95% CI 0.04–0.41). Thus, if all older adults with any sensory impairment had a moderate-to-rich social network or a moderate-to-high leisure activity, ∼22–25% of sensory impairment-related dementia cases could be prevented.

### Sensitivity analysis

In sensitivity analyses, we addressed possible reverse causality related to preclinical dementia at baseline. Cox models were repeated by excluding participants with incident dementia over the 3-year follow-up (*n* = 113) and/or baseline MMSE < 27 (*n* = 197). Estimates remained similar to those from the main analyses but were no longer significant, most likely due to loss of power (Supplementary Table 4).

## DISCUSSION

In this large-scale, population-based cohort study of dementia-free older adults with up to 12 years of follow-up, we found that 1) dual sensory impairment in vision and hearing was associated with higher risk of dementia, whereas single sensory impairment was not, and 2) the increased risk for dementia in people with dual sensory impairment may be attenuated by high leisure activity or a rich social network.

Several longitudinal population-based studies have reported a higher risk for dementia in individuals with either single or multiple sensory impairments [15, 33–36]. Our findings are in line with previous literature showing that dual or multiple sensory impairments are associated with a higher risk of dementia compared to single sensory impairments [32, 34–36]. However, there are inconsistencies in findings across previous studies. One recent study using self-reported measures of hearing and vision showed that self-reported hearing but not vision impairment was significantly associated with incident dementia, and that the combination of both impairments did not significantly predict incident dementia beyond the individual risks [37]. Another study including objective measures of hearing, vision, touch, and smell, found that the number of impairments was associated with an increased risk of dementia in a graded fashion [33]. The reasons for these inconsistencies may have to do with different approaches and methods of assessing the sensory function (i.e., objective versus self-reported) and variations in the cutoffs used to define the impairments.

Although a growing body of evidence suggests that sensory impairments may be linked to a higher risk of dementia and cognitive decline, not much has been done to explore the several hypotheses that attempt to explain this relationship. The complex intersection of processing sensory information and cognitive function makes it difficult to tease apart the mechanisms mediating the association between sensory impairment and dementia. One theory is that a decline in sensory function is part of the normal aging process and may therefore decline in concordance with cognitive function [38]. However, cognitive aging is very heterogeneous, with several risk and protective factors influencing its course. It has also been suggested that sensory function is related to Alzheimer’s disease pathology, with the strongest support from olfactory research [39]. Nevertheless, one study on multisensory impairments found that even after controlling for olfactory impairment, the additive effect of multiple sensory impairments persisted [33]. In the present study, we focused on the hypothesis that sensory impairments may lead to isolation and lack of engagement, and maintaining high levels in both could therefore play an important role in reducing the risk of dementia associated with sensory impairments.

Participating in leisure activities and having a rich social life has consistently been related to better cognitive outcomes in older adults [18, 20, 21]. Isolation and lack of engagement have been associated with depression and worse physical activity, and functional limitations which may, in turn, lead to accelerated cognitive decline [21]. Our findings support the hypothesis that a rich social network and engagement in leisure activities may buffer the effects of sensory impairment on the risk of dementia. This is in line with previous studies on risk factors for dementia, reporting a mitigating effect of leisure activity and social engagement in older adults with diabetes and cardio-metabolic disease [17, 18, 40]. Another possibility is that having a rich social network or high leisure activity could be a proxy for less severe impairment, thus future studies with more detailed sensory testing are warranted to understand this relationship.

Untangling the relationship between sensory impairment, dementia, and participation in leisure activity and social engagement is complex as several non-mutually exclusive explanations may exist. For instance, individuals with sensory impairment may have worse health in general and increased risk of mortality [12, 41], preventing them from staying active and socially engaged as well as contributing to a higher dementia risk. However, we adjusted for several indicators of health and functional status, which did not fully explain the reduced risk in participants with sensory impairment who had a rich social network or who were active. Another possible explanation may be that a lack of social connection and activity may lead to low mood or depression which may worsen the effects of sensory impairment on dementia. Indeed, previous studies found that concurrent sensory impairments were associated with poorer quality of life and increased risks of depressive symptoms [42, 43]. Adjusting for depression, however, did not modify the lower risk of dementia in participants with a rich social network or high leisure activity. A third possibility is that a “common cause”, such as structural and pathological processes in the brain, is causing both sensory and cognitive function to decline. For instance, the association of hearing impairment with regional brain atrophy over time was primarily observed in temporal lobe regions which are not only important for spoken language processing, but are also involved in semantic memory, sensory integration, and in the early stages of mild cognitive impairment or early dementia [44]. An early symptom of dementia can be apathy and social withdrawal [45, 46], therefore, we attempted to address this reverse causality by excluding participants who were diagnosed with dementia over the first 2-year follow-up and participants with MMSE scores below 27 at baseline, which produced similar results. However, to fully address the issue of reverse causation, longitudinal data starting in midlife or earlier is required. This would allow for a better understanding of whether sensory impairments are a cause for less social engagement and leisure activity, or if a lower engagement is an early indicator of dementia. A fourth possibility is that sensory impairments, if not adapted to or treated, may make it more difficult to stay socially connected and engaging in leisure activities, and that prolonged reductions in sensory input and stimulation lead to cognitive deterioration due to neuronal atrophy [47–49], Finally, a rich social network can provide a support system for older adults with sensory impairments in order to make better use of healthcare resources, self-management strategies and sensory aids, so that they can maintain a healthy lifestyle. Additional studies are necessary to determine the role of social network and leisure activities in the relationship between sensory impairment and dementia, particularly in more diverse populations.

This study has several strengths, including its longitudinal design with an extended period of observation and a very high rate of participant involvement. Additionally, it has a relatively substantial sample size and incorporates medical diagnoses made by physicians, data from national registries, and information on dementia status obtained from death certificates or medical records at the time of hospital discharge for participants who died during the follow-up. Moreover, we had detailed questionnaire data on social and lifestyle measures, allowing the possibility to assess the joint effects of leisure activities and social network on sensory impairment-related dementia risk. Furthermore, we performed sensitivity analyses to address the potential reverse causation by excluding participants with dementia during the first 3 years of follow up, and the findings were similar. However, some limitations need to be acknowledged. Our measure of hearing impairment was based on self-report and linkage with medical records, which may have resulted in underreporting of impairment. Vision impairment was based on a measure which only tested visual acuity and may therefore not have been sensitive to other forms of visual impairment. Such misclassifications may have biased our results toward the null, or toward more conservative estimates. Finally, the generalizability of the findings may be limited. The study population consisted of highly educated and relatively healthy older adults. This may have affected the magnitude of our results toward an underestimation.

In conclusion, our study demonstrated that dual sensory impairment is associated with higher risk of dementia, and that this risk might be buffered by having a rich social network and engaging in leisure activities. Our results highlight the need for future behavioral interventions that integrate mental, social, and physical aspects of lifestyle to investigate how and to what extent dementia can be prevented in people with sensory impairments. Our findings also call for a better awareness, monitoring and control of sensory impairments by professional and informal care providers of older people.

## Supplementary Material

Supplementary Material

## Data Availability

The SNAC-K project is a population-based study on aging and dementia (http://www.snac-k.se/). Access to these original data is available to the research community upon approval by the SNAC-K data management and maintenance committee. Applications for accessing these data can be submitted to Maria Wahlberg (Maria.Wahlberg@ki.se) at the Aging Research Center, Karolinska Institutet.
