# Supplementary material for: Association Between Sensory Impairment and Dementia: The Roles of Social Network and Leisure Activity
Source: J Alzheimers Dis. 2023 Jul 18;94(2):585–95. doi: 10.3233/JAD-230041 (PMC10357179; doi:10.3233/JAD-230041)
Supplement: Supplementary Material [file jad-94-jad230041-s001.pdf]

Supplementary Material

Association Between Sensory Impairment and Dementia: The Roles of Social Network and Leisure Activity

Supplementary Material 1. Leisure activities interview protocol Instructions: Following are questions regarding your leisure activities. Write or check the answer that best suits you.

Have you participated in any of the following entertainment or cultural activities in the last 12 months?	
1. Cinema/Theatre/Concert<br>2. Sporting Events<br>3. Museum/Art exhibit<br>4. Go to Restaurant/Pub/Café<br>5. Bingo<br>6. Dancing<br>7. Attend church/revival meeting<br>8. Participate in study circle or a course<br>9. Participate in volunteer work<br>10. Participate in association/club work<br>11. Travel<br>12. Gardening<br>13. Hiking in forest/picking berries, mushrooms<br>14. Hunting, fishing<br>15. Knitting, weaving, sewing<br>16. Painting, drawing, working with clay/pottery<br>17. Home repairs<br>18. Car or mechanical repairs<br>19. Read the newspaper/ magazine or journal/books<br>20. Watch TV<br>21. Play chess/card games<br>22. Play musical instrument<br>23. Listen to music<br>24. Use the internet or play computer games	For each item:<br>Yes, to the same degree<br>Yes, to a higher degree<br>Yes, but to a lesser degree<br>No<br>No response

In the last 12 months:	
25. Did you regularly engage in light exercise (e.g., walking along roads or in parks, walking in the woods, short bicycle rides, light aerobics, golf)?	Every day; Several times a week; 2-3 times/month; Less; Never; Don't know; No response; Several options checked
26. Did you regularly engage in moderate to intense exercise, now or previously (e.g., jogging, long power walks, heavy-duty gardening, long bicycle rides, high-intensity aerobics, long distance ice skating, swimming, ball sports (not golf) or other similar activity)?	Every day; Several times a week; 2-3 times/month; Less; Never; Don't know; No response; Several options checked

Supplementary Material 2. Social network questionnaire (Hanson et al. 1997)
Instructions: Following there are questions regarding your social network. Write or check the answers that best suit you.

Frequency of direct or remote contacts

How often do you meet the following in person?	
1. Parents<br>2. Children<br>3. Son/daughter-in-law<br>4. Grandchildren<br>5. Siblings<br>6. Other relative<br>7. Neighbor<br>8. Friend	For each item:<br>Daily, more than twice/week<br>Weekly, more than twice/month<br>Monthly, more than 6 times/year<br>Quarterly, more than once/year<br>Less often<br>Never<br>Don't know<br>N/A (e.g., have no children or parent no longer alive)<br>No response<br>Several options checked

How often are you in touch, via telephone, letters, e-mail with the following:	
9. Parents<br>10. Children<br>11. Son/daughter-in-law<br>12. Grandchildren<br>13. Siblings<br>14. Other relative<br>15. Neighbor<br>16. Friend	For each item:<br>Daily, more than twice/week<br>Weekly, more than twice/month<br>Monthly, more than 6 times/year<br>Quarterly, more than once/year<br>Less often<br>Never<br>Don't know<br>N/A (e.g., have no children or parent no longer alive)<br>No response<br>Several options checked

Satisfaction with these contacts

How often are you in touch, via telephone, letters, e-mail with the following:	
17. Parents<br>18. Children<br>19. Son/Daughter-in-law<br>20. Grandchildren<br>21. Siblings<br>22. Other relative<br>23. Neighbor<br>24. Friend	For each item:<br>Yes<br>No<br>Don't know<br>N/A (e.g., do not have children or parents no longer alive)<br>No response<br>Several options checked

Perceived material support

27. Could you get help from one or several people in case of illness or other practical troubles? (e.g., borrow little things, get help with repairs, get advice and information)<br>28. Do you know one or several people who could help you write an official letter or appeal a government decision?	For each item:<br>Yes, without a doubt<br>Yes, probably<br>No, probably not<br>No, not at all<br>Don't know<br>No response<br>Several options checked

Social network size

29. How many people do you feel you know well and can talk to about most things? (e.g., relatives, friends, neighbors, and/or colleagues)	None; 1-2 people; 3 people; 4-6 people; 7-9 people; 10-15 people; 16-30 people; More than 30 people; Don't know; No response; Several options checked

Perceived psychological support

30. Do you feel that you know one or a few people who could give you proper personal/emotional support to manage the stress and troubles of life?<br><br>31. Do you know someone with whom you can be yourself, who accepts you for all your good and bad qualities?	For each item:<br>Yes, without a doubt<br>Yes, probably<br>No, probably not<br>No, not at all<br>Don't know<br>No response<br>Several options checked

Sense of affinity with association members

32. If you are part of an association/organization, would you say you feel a strong sense of belonging to this group and its members?	I am not member of any association; To a high degree; To a modest degree; Not especially; Not at all; Don't know; No response; Several options checked

Sense of affinity with relatives

33. Do you feel a strong sense of kinship with your relatives (beyond spouse/partner/children)?	Have no family; To a high degree; To a modest degree; Not especially; Not at all; Don't know; No response; Several options checked

Sense of affinity with residence area

34. Are you rooted in and feel a strong sense of kinship with your neighborhood?	To a high degree; To a certain degree; Not especially; Not at all; Don't know; No response; Several options checked

Being part of a group of friends

35. Are you part of a group of friends/acquaintances who have something in common or do some activity together (e.g., play cards, listen to music, go on excursions, etc.)?<br>36. Are you part of a group of friends/acquaintances who have something in common or do some activity together (e.g., play cards, listen to music, go on excursions, etc.)?	Yes; No; Don't know; No response; Several options checked<br><br><br><br>Yes; No; Don't know; No response; Several options checked

Supplementary Table 1. Incidence rates (IR) per 1000 person-years and hazard ratios (HR) with 95% CI of incident dementia (n=381) by social network and leisure activity (N=2579).

	No. events/ person-year	IR (95% CI)	HR (95% CI)[a]	HR (95% CI)[b]
Social network				
Low	136/5260	25.86 (21.86 to 30.59)	Reference	Reference
Moderate	124/7301	16.98 (14.24 to 20.25)	0.81 (0.61 to 1.06)	0.68 (0.52 to 0.88)
High	85/8460	10.10 (8.12 to 12.42)	0.52 (0.38 to 0.71)	0.40 (0.29 to 0.54)
Leisure activity				
Low	119/4988	23.86 (19.93 to 28.55)	Reference	Reference
Moderate	126/9271	13.59 (11.41 to 16.18)	0.68 (0.52 to 0.89)	0.55 (0.42 to 0.72)
High	41/5612	7.31 (5.38 to 9.92)	0.39 (0.26 to 0.59)	0.29 (0.20 to 0.42)

[a] Model 1, adjusted for baseline age, sex, and education.

[b] Model 2, adjusted additionally for smoking, alcohol consumption, ADL limitations, diabetes, depression, cardiovascular disease, and cerebrovascular disease.

Stratified analyses by *APOE* ε4 status and age.

Supplementary Table 2. Hazard ratios (HR) with 95% CI of incident dementia (n=381) by sensory impairment status stratified by *APOE* ε4 status (N=2579).

	APOE ε4 negative		*APOE* ε4 positive	
Visual and hearing impairment	HR (95% CI)[a]	HR (95% CI)[b]	HR (95% CI)[a]	HR (95% CI)[b]
None	Reference	Reference	Reference	Reference
Any	1.69 (1.24 to 2.29)	1.54 (1.13 to 2.09)	0.81 (0.55 to 1.19)	0.68 (0.46 to 1.00)
Vision only	1.72 (1.14 to 2.62)	1.55 (1.02 to 2.36)	0.62 (0.33 to 1.15)	0.52 (0.28 to 0.98)
Hearing only	1.27 (0.86 to 1.88)	1.26 (0.85 to 1.86)	0.87 (0.57 to 1.33)	0.75 (0.48 to 1.18)
Dual	2.40 (1.63 to 3.53)	2.03 (1.35 to 3.06)	1.13 (0.56 to 2.28)	0.91 (0.45 to 1.87)

APOE ε4: Apolipoprotein epsilon 4.

[a] Model 1, adjusted for baseline age, sex, and education.

[b] Model 2, adjusted additionally for smoking, alcohol consumption, ADL limitations, diabetes, depression, cardiovascular disease, and cerebrovascular disease.

Supplementary Table 3. Hazard ratios (HR) with 95% CI of incident dementia (n=381) by sensory impairment status stratified by baseline age group (N=2,579).

Visual and hearing impairment	Age<78 (n=1,495)		Age≥78 (n=1084)	
	HR (95% CI)[a]	HR (95% CI)[b]	HR (95% CI)[a]	HR (95% CI)[b]
None	Reference	Reference	Reference	Reference
Any	0.89 (0.58 to 1.37)	0.90 (0.59 to 1.40)	1.46 (1.12 to 1.90)	1.32 (1.01 to 1.73)
Vision only	1.06 (0.59 to 1.91)	1.13 (0.62 to 2.09)	1.28 (0.92 to 1.77)	1.13 (0.79 to 1.62)
Hearing only	0.57 (0.31 to 1.07)	0.30 (1.05 to 1.06)	1.27 (0.92 to 1.77)	1.26 (0.90 to 1.75)
Dual	2.04 (1.02 to 4.07)	2.04 (0.98 to 4.25)	2.00 (1.44 to 2.79)	1.69 (1.19 to 2.39)

[a] Model 1, adjusted for baseline age, sex, and education.

[b] Model 2, adjusted additionally for smoking, alcohol consumption, ADL limitations, diabetes, depression, cardiovascular disease, and cerebrovascular disease.

Supplementary Material 3. Sensitivity analysis

In sensitivity analyses, we first addressed possible reverse causality related to preclinical dementia at baseline. Cox models were repeated by excluding participants with incident dementia over 3-year follow-up (n=113) and baseline MMSE≤27 (n=197). Results remained similar to that from the initial analyses (Supplementary Table 4).

Supplementary Table 4. Hazard ratios (HRs) and 95% CIs of the effect of sensory impairment and active life on dementia in the SNAC-K dementia-free cohort after excluding participants with incident dementia at 3-year follow-up (n=113) and Mini-Mental State Examination ≤27 (n=197).

Joint effect		n	HR (95% CI)[a]	HR (95% CI)[b]
Social network	Sensory impairment			
Moderate-to-high	None	909	Reference	Reference
Low	None	268	1.42 (0.91 to 2.22)	1.46 (0.94 to 2.26)
Moderate-to-high	Single		1.11 (0.78 to 1.59)	1.06 (0.74 to 1.52)
Low	Single		1.57 (1.00 to 2.45)	1.49 (0.94 to 2.36)
Moderate-to-high	Dual	112	1.39 (0.78 to 2.47)	1.15 (0.77 to 1.73)
Low	Dual	78	1.92 (0.93 to 3.97)	1.85 (0.88 to 3.89)
Leisure Activity				
Moderate-to-high	None	874	Reference	Reference
Low	None	262	1.81 (1.19 to 2.73)	1.78 (1.18 to 2.69)
Moderate-to-high	Single		1.11 (0.75 to 1.62)	1.07 (0.73 to 1.58)
Low	Single		1,56 (0.99 to 2.48)	1.39 (0.85 to 2.28)
Moderate-to-high	Dual	105	1.28 (0.70 to 2.34)	1.19 (0.62 to 2.26)
Low	Dual	59	3.10 (1.35 to 7.11)	2.58 (1.08 to 6.14)

[a] Model 1, adjusted for baseline age, sex, education, and baseline MMSE.

[b] Model 2, adjusted for Model 1 + smoking, alcohol consumption, cardiovascular disease, cerebrovascular disease, and diabetes.
